# Elevated Carbon Dioxide Increases Contents of Flavonoids and Phenolic Compounds, and Antioxidant Activities in Malaysian Young Ginger (*Zingiber officinale* Roscoe.) Varieties

**DOI:** 10.3390/molecules15117907

**Published:** 2010-11-03

**Authors:** Ali Ghasemzadeh, Hawa Z.E. Jaafar, Asmah Rahmat

**Affiliations:** 1 Department of Crop Science, Faculty of Agriculture, University Putra Malaysia, 43400 UPM Serdang, Selangor, Malaysia; E-Mail: upmali@yahoo.com (A.G.); 2 Department of Nutrition & Dietetics, Faculty of Medicine & Health Sciences, University Putra Malaysia, 43400 UPM Serdang, Selangor, Malaysia; E-Mail: asmah@medic.upm.edu.my (A.R.)

**Keywords:** CO_2_ enrichment, flavonoids, phenolic acids, free radical scavenging (DPPH) power

## Abstract

*Zingiber officinale* Roscoe. (Family Zingiberaceae) is well known in Asia. The plant is widely cultivated in village gardens in the tropics for its medicinal properties and as a marketable spice in Malaysia. Ginger varieties are rich in physiologically active phenolics and flavonoids with a range of pharmacological activities. Experiments were conducted to determine the feasibility of increasing levels of flavonoids (quercetin, rutin, catechin, epicatechin, kaempferol, naringenin, fisetin and morin) and phenolic acid (gallic acid, vanillic acid, ferulic acid, tannic acid, cinnamic acid and salicylic acid), and antioxidant activities in different parts of Malaysian young ginger varieties (Halia Bentong and Halia Bara) with CO_2_ enrichment in a controlled environment system. Both varieties showed an increase in phenolic compounds and flavonoids in response to CO_2_ enrichment from 400 to 800 µmol mol^-1^ CO_2_. These increases were greater in rhizomes compared to leaves. High performance liquid chromatography (HPLC) results showed that quercetin and gallic acid were the most abundant flavonoid and phenolic acid in Malaysian young ginger varieties. Under elevated CO_2_ conditions, kaempferol and fisetin were among the flavonoid compounds, and gallic acid and vanillic acid were among the phenolic compounds whose levels increased in both varieties. As CO_2_ concentration was increased from 400 to 800 µmol mol^-1^, free radical scavenging power (DPPH) increased about 30% in Halia Bentong and 21.4% in Halia Bara; and the rhizomes exhibited more enhanced free radical scavenging power, with 44.9% in Halia Bentong and 46.2% in Halia Bara. Leaves of both varieties also displayed good levels of flavonoid compounds and antioxidant activities. These results indicate that the yield and pharmaceutical quality of Malaysian young ginger varieties can be enhanced by controlled environment production and CO_2_ enrichment.

## 1. Introduction

The increase of atmospheric CO_2_ due to global climate change and/or horticultural practices has direct and indirect effects on secondary metabolite synthesis in plants [1]. The responses observed in different plants show a wide range of patterns, either in the structure of primary and secondary metabolites or in the biomass production. These kinds of responses may occur in natural plant ecosystems, but may also be created deliberately by deliberate CO_2_ enrichment techniques in controlled environment systems to increase the production of some plants and some secondary compounds [2,3,4]. In the previous and current century, there has been an increase in the use of herbal products for traditional medicine and foods, which is attributable to a new way of life that is related to arising interest in the use of natural products. At the same time, there is also a keen interest in functional foods, which in some cases have shown little distinction between herbs and spices as a wide range of natural products are mostly produced from herbs or medicinal plants. They have been introduced to society as part of traditional cultural practices, and hence, there are only very soft rules existing about their commercial regulation [5]. 

Crops under enriched CO_2_ atmospheres acquire positive features with enhanced plant adaptation and growth. The greatest advantages of CO_2_ enrichment is in the enhancement of photosynthetic capacity, particularly under adverse climatic conditions and this would becomes most apparent in the vegetative growth of young plants [6,7]. Rising levels of atmospheric CO_2_ can alter plant growth and partitioning of secondary metabolites [8]. This can be proved by the results of the study of Wang *et al.* [9] showing that elevated CO_2_ concentrations in the atmosphere enhanced vegetative growth, carbohydrate accumulation, and fruit productivity in strawberry. It is well established that environmental factors can influence the production of secondary metabolites in plants [10]. For example, under nutrient deficient conditions, the levels of non-nitrogenous metabolites derived from the shikimic acid pathway such as phenolic acids, lignin, hydrolysable tannins, and proanthocyanidins usually increase in woody plants. The increase in C-based secondary metabolites frequently occurs when environmental conditions also promote an accumulation of non-structural carbohydrates (TNC) in plants. Elevated atmospheric CO_2_ concentrations often increase TNC concentrations in plants and possibly stimulate secondary metabolism [11].

Flavonoids belong to a large family of polyphenolic components synthesized by plants [12]. High contents of natural phenolic acids and flavonoids are found in green tea, fruits, and vegetables, while some amounts of phenolics exist in red wine and coffee [13,14]. Free radicals and single oxygen are recognized as major factors causing various chronic diseases such as cancer, diabetes, *etc.* Therefore, according to recent and previous studies, the health maintenance function of antioxidant components in various foods has received much attention in recent years [15,16]. Phenolic acids and flavonoids are antioxidants with health benefits such as anti-inflammatory and antitumor effect [17,18,19,20]. Sung-jin *et al.* [21] showed that some flavonoid components in green tea are effective in inhibiting cancer or induce mechanisms that may kill cancer cells and inhibit tumor invasion. 

*Zingiber officinale* is one of the traditional folk medicinal plants that have been used by Polynesians for over 2,000 years for treating diabetes, high blood pressure, cancer, fitness and many other illnesses [22]. The use of ginger for food and cooking has a long history in Asia. *Zingiber officinale* contains a number of antioxidants such as ascorbic acid, and phenolic acids [23]. Easily cultivable, *Zingiber officinale*, with its wide range of antioxidants, can be a major source of natural or phytochemical antioxidants [24]. Although various extracts are obtained from ginger, it is the CO_2_ extracts that are richest in polyphenol compounds and have a composition that closely resembles that of the rhizomes [25,26].

Malikov *et al.* [30] reviewed the biosynthesis and properties of phenolic and flavonoid compounds detected from *Scutellaria* species. Increased concentration of flavonoids through CO_2_ enrichment has the potential to enhance the production and quality of medicinal plants such as *Scutellaria*. Increases in the levels of phenolic and flavonoid components of *Populus tremuloides* by CO_2_ enrichment method has been reported by Lindroth *et al.* [31]. 

The main objectives of this study were to evaluate the effects of carbon dioxide enrichment on concentration of phenolics and flavonoids compound in extracts of young ginger (*Zingiber officinale)* varieties (Halia Bentong and Halia Bara), and to determine the antioxidant activity.

## 2. Results and Discussion

### 2.1. HPLC Analysis of Flavonoids

The results obtained from the preliminary analysis of flavonoids are shown in Table 1. Increasing the CO_2_ concentration from 400 to 800 µmol mol^-1^ resulted in enhanced quercetin, catechin, kaempferol and fisetin levels in the leaves and rhizomes of both varieties, and increase in naringenin content in just the leaves. On the other hand, the contents of rutin, epicatechin and morin decreased in ginger parts with rising of CO_2_ concentration from ambient to 800 µmol mol^-1^. It can be seen from the data in this table that quercetin content in ginger, when compared with other plants, for example red chilli (0.799 mg g^-1^ DW), bird chilli (0.392 mg g^-1^ DW), bell pepper (0.448 mg g^-1 ^DW), black tea (1.107 mg g^-1^ DW), onion (1.49 mg g^-1^ DW) and semambu (1.18 mg g^-1^ DW) [34] registered substantially high levels in both the leaves (1.33 mg g^-1 ^DW) and rhizomes (1.27 mg g^-1 ^DW) of Halia Bara exposed to elevated CO_2_ concentration at 800 µmol mol^-1^. 

**Table 1:** The concentrations of some flavonoids compounds in two varieties of *Zingiber officinale,* Halia Bentong (a) and Halia Bara (b) grown under different CO_2_ concentrations.

Flavonoid compounds	(a) Halia Bentong			
	400	800		
	Leaves	Rhizomes	Leaves	Rhizomes
Quercetin	0.972 ± 0.013c	0.895 ± 0.039c	1.22 ± 0.07b	1.138 ± 0.023b
Rutin	0.171 ± 0.0028de	0.452 ± 0.004a	0.141 ± 0.031e	0.388 ± 0.026b
Epicatechin	0.122 ± 0.018a	0.083 ± 0.007bc	0.073 ± 0.008c	0.048 ± 0.018d
Catechin	0.409 ± 0.027d	0.491 ± 0.019cd	0.673 ± 0.044ab	0.637 ± 0.034b
Kaempferol	0.042 ± 0.002e	0.053 ± 0.003de	0.118 ± 0.014c	0.148 ± 0.023b
Naringenin	0.089 ± 0.0052c	0.047 ± 0.003d	0.127 ± 0.022b	0.083 ± 0.004c
Fisetin	0.986 ± 0.012e	0.633 ± 0.033f	2.05 ± 0.27c	2.82 ± 0.19a
Morin	0.514 ± 0.027e	0.463 ± 0.014e	0.49 ± 0.052e	0.875 ± 0.036a
**Flavonoid compounds**	**(b) Halia Bara**			
	**400**		**800**	
	**Leaves**	**Rhizomes**	**Leaves**	**Rhizomes**
Quercetin	1.19 ± 0.122ab	0.986 ± 0.032c	1.33 ± 0.134a	1.27 ± 0.01a
Rutin	0.174 ± 0.007d	0.334 ± 0.009c	0.151 ± 0.025de	0.404 ± 0.016b
Epicatechin	0.12 ± 0.004a	0.103 ± 0.0035ab	0.096 ± 0.022bc	0.037 ± 0.009d
Catechin	0.668 ± 0.079ab	0.533 ± 0.034c	0.733 ± 0.014a	0.682 ± 0.05ab
Kaempferol	0.051 ± 0.002de	0.068 ± 0.005d	0.163 ± 0.011ab	0.181 ± 0.009a
Naringenin	0.061 ± 0.004d	0.028 ± 0.003e	0.155 ± 0.027a	0.121 ± 0.011b
Fisetin	1.53 ± 0.121d	1.32 ± 0.12d	2.38 ± 0.395b	3.11 ± 0.185a
Morin	0.765 ± 0.024b	0.606 ± 0.006d	0.661 ± 0.029c	0.515 ± 0.025e

All analyses are the mean of triplicate measurements ± standard deviation. Results expressed in mg g^-1^ of dry plant material. Means not sharing a common letter were significantly different at P ≤ 0.05.

Kaempferol is a rare flavonoid component in plants, but it was detected in the leaves (0.042–0.163 mg g^-1^ DW) and rhizomes (0.053–0.181 mg g^-1^ DW) of both Halia Bara and Halia Bentong. These contents were slightly higher than those recorded in green chilli (0.039 mg g^-1^ DW), sengkuang (0.037 mg g^-1^ DW), white radish (0.0383 mg g^-1^ DW), and pegaga (0.0205 mg g^-1^ DW). Nevertheless, both ginger varieties had lower kaempferol contents when compared to cekur manis (0.323 mg g^-1^ DW), pumpkin (0.371 mg g^-1^ DW), and carrot (0.140 mg g^-1^ DW) [35]. Tolonen *et al.* [36] reported very low kaempferol contents (9 mg/g DW) in white cabbages, and it was the only flavonoid found. Meanwhile, Kim [37] detected about 0.1–0.8 mg/g fm of quercetin and kaempferol aglycone contents in green cabbages. Exposing ginger plants to 800 µmol mol^-1^ of CO_2_ concentration saw the synthesis of kaempferol enhanced to 0.163 and 0.181 mg g^-1^ DW in Halia Bara leaves and rhizomes, respectively.

Fisetin is another rare yet well known flavonoid component in plants. Previous studies showed that fisetin had anti-inflammatory [35,38], anti-carcinogenic [39] and strong antioxidant [39] effects. Ginger leaves and rhizomes exhibited good potential levels of this flavonoid. It seemed that fisetin content could also be improved by increasing CO_2_ concentration in both of varieties, especially in Halia Bara (leaves: 1.53 increased to 2.38 mg g^-1^ DW; rhizome: 1.32 increased to 3.11 mg g^-1^ DW). Morin is another flavonoid belonging to the flavonols group. Morin acts as a chemopreventive agent *in vitro* and *in vivo* against oral carcinogenesis [40,41]. The importance of morin and related compounds as anti-tumour drugs has also been widely recognized [42]. In comparison with old fustic (*Chlorophora tinctoria*), osage orange (*Maclura pomifera*) [43], almonds (*P. guajava* L.) [44], mill (*Prunus dulcis*), fig (*Chlorophora tinctoria*) [43], onion and apple [44], both the leaves and rhizomes local ginger varieties showed good levels of morin when grown under both 400 and 800 µmol mol^-1^ CO_2_ conditions, indicating that the plant is naturally a good source of morin, although the content of the latter was variable. For example, the content of morin in the leaves decreased in both varieties with increasing of CO_2_ concentration, while a high content of morin (0.875 *vs.* 0.463 mg g^-1^ DW) was obtained from extract of Halia Bentong rhizomes grown under elevated CO_2_. 

Similar trends of increasing concentration of flavonoid components with increasing CO_2_ concentration was observed in *Betula pendula* and strawberry [2,9]. Wang *et al.* [9], reported growing strawberry plants under CO_2_ enrichment conditions (950 µmol mol^-1^) significantly enhanced fruit *p*-coumaroylglucose, dihydroflavonol, quercetin 3-glucoside, quercetin 3-glucuronide, and kaempferol 3-glucoside contents, as well as cyanidin 3-glucoside, pelargonidin 3-glucoside, and pelargonidin 3-glucoside succinate content. This finding is in agreement with Sttute *et al.* [32] who showed the ability of elevated CO_2_ concentrations to enhance flavonoid components (apygenin, baicalin, scutellarein) in *Scutellaria *species. The percentages of increase or decrease in flavonoid contents of ginger when exposed to 800 µmol mol^-1^ concentrations of CO_2 _are tabulated in Table 2. 

**Table 2:** Percent of increase or decrease of flavonoid compounds in two varieties of *Zingiber officinale* grown under elevated CO_2_ concentration (800 µmol mol^-1^).

Flavonoid compounds	Halia Bentong		Halia Bara	
	Leaves	Rhizomes	Leaves	Rhizomes
Quercetin	+25.5	+27.2	+9.2	+28.8
Rutin	-17.5	-14.2	-13.2	+21.0
Epicatechin	-40.2	-42.2	-20.0	-64.1
Catechin	+64.5	+29.7	+9.7	+28.0
Kaempferol	+181.0	+179.2	+219.6	+166.2
Naringenin	+42.7	+76.6	+154.1	+332.1
Fisetin	+107.9	+345.5	+55.6	+135.6
Morin	-4.7	+89.0	-13.6	-15.0

Results expressed in percent; + and – indicate respectively increases and decreases of component concentrations when exposed to CO_2_.

**Figure 1:**
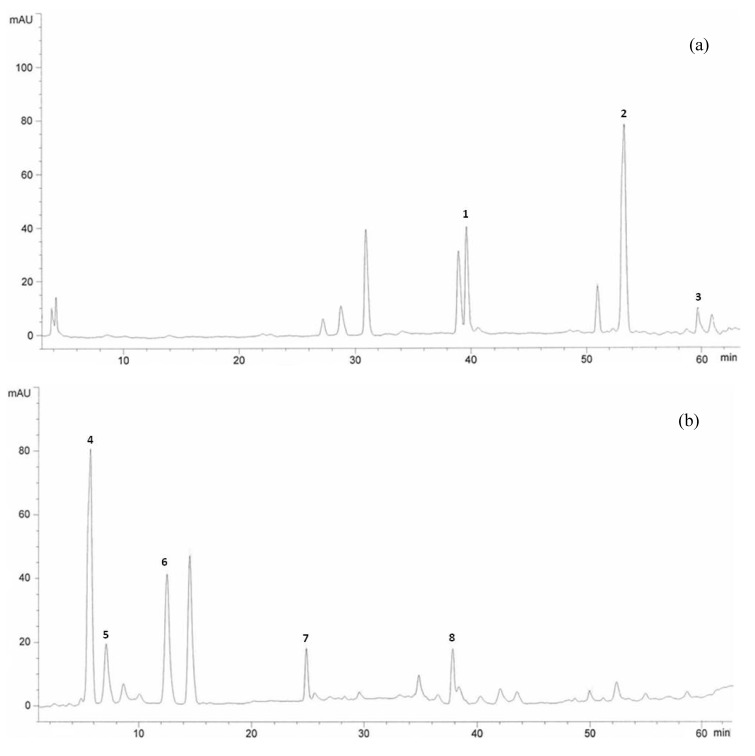
HPLC chromatogram of Halia Bentong ginger (Zingiber officinale) leaves extracts at wavelengths of 360 nm (a), and 280 nm (b). Identification of compounds: quercetin (1), rutin (2), kaempferol (3), fisetin (4), morin (5), catechin (6), epicatechin (7), naringenin (8).

According to data from this table, kaempferol levels were more enhanced in varieties grown under elevated carbon dioxide conditions and after that, fisetin and naringenin were more enhanced. On average flavonoid compounds increased 44.9% in leaves and 86.3% in rhizomes of Halia Bentong and 50.1 % in leaves and 79% in rhizomes of Halia Bara when exposed to elevated carbon dioxide conditions. To the best of our knowledge the current study is the first report of fisetin, morin and naringenin in young ginger varieties. Figure 1 shows the HPLC chromatogram of flavonoids analysis in Halia Bentong extract (leaves).

### 2.2. HPLC Analysis of Phenolic Compounds

Like the alteration of flavonoid accumulation in both varieties of ginger when they were exposed to 400 to 800 µmol mol^-1^ CO_2_ the phenolic contents also increased in the leaves more than in the rhizomes. Phenolic contents are influenced by the interaction between varieties and parts of the plants. Partitioning and accumulation of phenolics in different parts of ginger grown under ambient CO_2_ followed the trend of leaves > rhizomes. Rhizomes in both of varieties had more phenolic content when exposed to elevated CO_2_. Among the phenolic acid compounds, gallic acid had a higher content in both ginger varieties (Table 3). Elevated CO_2_ had significant effects (p ≤ 0.001) on the synthesis of phenolics. What is interesting in this data is that vanillic acid, cinnamic acid and salicylic acid were not detected in ginger grown under ambient (400 µmol mol^-1^) conditions. Conversely, tannic acid was not detected in gingers grown under elevated CO_2_ (800 µmol mol^-1^). Results imply that different CO_2_ concentrations have variable effects on each of the phenolic components. Among the studied phenolic compounds vanillic acid, cinnamic acid and salicylic acid were not detected in Halia Bentong grown under ambient CO_2_. Also cinnamic acid and salicylic acid were not detected in Halia Bara grown under ambient CO_2_. Tannic acid was not detected in those varieties grown under elevated (800 µmol mol^-1^) CO_2_. 

**Table 3:** The concentrations of some phenolics compounds in two varieties of *Zingiber officinale,* Halia Bentong (a) and Halia Bara (b) grown under different CO_2_ concentrations.

Phenolic compounds	(a) Halia Bentong			
	400		800	
	Leaves	Rhizomes	Leaves	Rhizomes
Gallic acid	0.173 ± 0.0091d	0.141 ± 0.031d	0.576 ± 0.049b	0.489 ± 0.043c
Vanillic acid	nd	nd	0.229 ± 0.058b	0.335 ± 0.028a
Ferulic acid	0.081 ± 0.022f	0.116 ± 0.016ef	0.117 ± 0.026de	0.21 ± 0.022b
Tannic acid	0.388 ± 0.072a	nd	nd	nd
Cinnamic acid	nd	nd	0.134 ± 0.027a	0.0336 ± 0.255b
Salicylic acid	nd	nd	0.22 ±0.021b	0.037 ± 0.0125c
**Phenolic compounds**	**(b) Halia Bara**			
	**400**		**800**	
	**Leaves**	**Rhizomes**	**Leaves**	**Rhizomes**
Gallic acid	0.191±0.008d	0.152+0.0081d	0.645±0.066a	0.537±0.034bc
Vanillic acid	0.082±0.016c	nd	0.24±0.052b	0.357±0.038a
Ferulic acid	0.071±0.017f	0.148+0.017cd	0.162±0.014c	0.285±0.038a
Tannic acid	0.224±0.041b	nd	nd	nd
Cinnamic acid	nd	nd	0.125±0.027a	0.0457±0.01b
Salicylic acid	nd	nd	0.269±0.027a	0.0417±0.044c

All analyses are the mean ± standard deviation (N = 2). Results expressed in mg g^-1^ of dry plant material. nd: non detected. Means not sharing a common letter in each row (a:H.Bentong and b: H. Bara) were significantly different at P ≤ 0.05.

**Figure 2:**
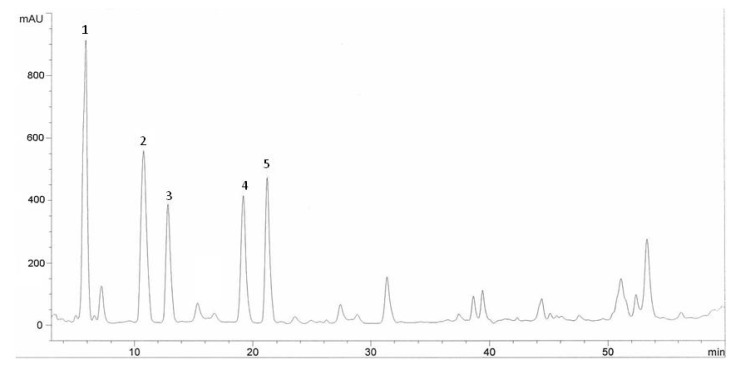
HPLC chromatogram of Halia Bentong ginger (*Zingiber officinale*) leaves extracts. Identification of compounds: gallic acid (1), vanillic acid (2), ferulic acid (3), cinnamic acid (4), salicylic acid (5).

Salicylic acid belongs to plant phenolics group and is found in some plant species, with the highest levels being observed in the inflorescences of thermogenic plants and in spice herbs [45]. A high content of salicylic acid (0.269 mg g^-1^ DW) was detected in extract of Halia Bara leaves grown under 800 µmol mol^-1^ CO_2_. The results of previous studies showed that salicylic acid could enhance plant growth and yield. Jeyakumar *et al.* [46] reported that salicylic acid was able to enhance the dry matter production in black gram, while Nagasubramaniam *et al.* [45] stated that salicylic acid increased plant height, leaf area, crop growth rate, and total dry matter production in baby corn. Salicylic acid was able to enhance plant growth by improving nutrition uptake. According to previous studies we could say that increasing of cinnamic acid in ginger might be one of the reasons for increased ginger growth under elevated carbon dioxide. 

**Table 4:** Percent of increase or decrease of phenolic compounds in two varieties of *Zingiber officinale* grown under elevated CO_2_ concentration (800 µmol mol^-1^).

Phenolic compounds	Halia Bentong		Halia Bara	
	Leaves	Rhizomes	Leaves	Rhizomes
Gallic acid	+232.4	+246.8	+252.4	+262.8
Vanillic acid	+100	+100	+192.6	+100
Ferulic acid	+44.4	+81	+128.2	+92.5
Tannic acid	-100	0	-100	0
Cinnamic acid	+100	+100	+100	+100
Salicylic acid	+100	+100	+100	+100

Results expressed in percent; + and – indicate respectively increase and decrease of component concentrations when exposed to CO_2_.

According to data from Table 4, gallic acid more enhanced in those varieties grown under elevated carbon dioxide conditions and after that vanillic acid and ferulic acid were more enhanced. On average phenolic compounds increased 79.4% in leaves and 107.6% in rhizomes of Halia Bentong and 112.2% in leaves and 109.2% in rhizomes of Halia Bara when exposed to elevated carbon dioxide conditions. 

### 2.3. Radical Scavenging Activity (DPPH)

**Figure 3:**
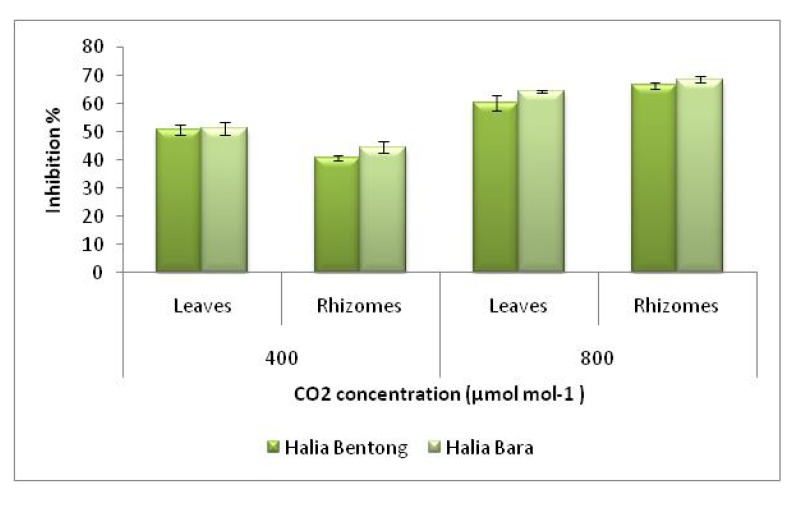
DPPH scavenging activities of the methanolic extracts in different parts of two varieties of *Zingiber officinale* (error bar represents standard deviation).

**Figure 4:**
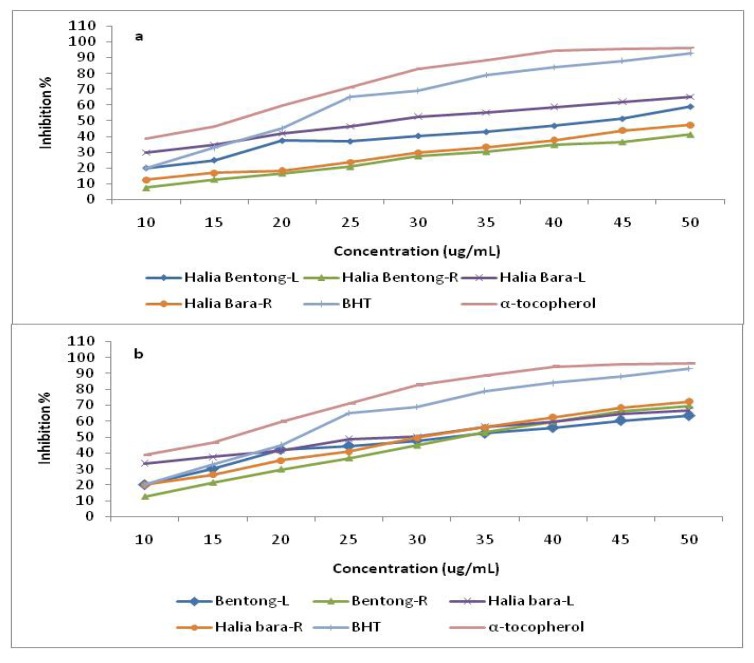
DPPH radical scavenging activity of the methanolic extracts in different parts of two varieties of *Zingiber officinale *compared with positive controls butylated hydroxytoluene (BHT) and α-tocopherol. L and R represent the leaves and rhizomes at 400 µmol mol^-1^ CO_2_ (a), and 800 µmol mol^-1^ CO_2 _(b).

Under ambient conditions (400 µmol mol^-1^ CO_2_), ginger leaves exhibited higher radical scavenging activity than rhizomes (Figure 3). At a concentration of 30 µg mL^-1^ leaves of Halia Bara showed a 50.0% inhibition of free radicals, and at 45 µg mL^-1^, the scavenging activity of the methanolic extract of Halia Bara leaves grown under ambient CO_2_ concentration (400 µmol mol^-1^) reached 62.1%, while at the same extract concentration, that of the rhizomes was 42.0% (Figure 4), while 50% free radical scavenging activity was observed for Halia Bentong leaves at 45 µg mL^-1 ^extract concentration, implying that Halia Bara, under natural environmental conditions, had higher antioxidant properties than Halia Bentong, and that was found in the leaves. 

The effect of antioxidants on DPPH scavenging is due to their hydrogen donating ability. The results of the current study showed that DPPH radical scavenging abilities of the extracts of ginger parts were less than those of butylated hydroxytoluene (BHT) (83.7%) and α-tocopherol (92.3%) at 45 µg mL^-1^. Antioxidant activity in the leaves and rhizomes were enhanced by increasing the CO_2_ concentration (Figure 3). When the CO_2_ concentration was increased from 400 to 800 µmol mol^-1^, the free radical scavenging power increased about 30.0% in Halia Bentong and 21.4% in Halia Bara. When comparing the ginger parts, it was found that the free radical scavenging power was more enhanced in the rhizomes than in the leaves (44.9% in Halia Bentong; 46.2% in Halia Bara). The above results also suggested that Halia Bentong seemed to be more responsive to increased CO_2_ concentration than Halia Bara, although the rhizomes of the latter were more receptive to elevated CO_2_ in the improvement of the antioxidative power. At low concentrations (10 and 15 µg mL^-1^) the DPPH activities of Halia Bara leaves was higher (35% and 38%, respectively) than BHT (20% and 32%, respectively). The DPPH scavenging activities of rhizomes in both varieties also increased after the concentration was increased to 35 µg mL^-1^, and these activities were higher than those recorded from the leaves using the same concentration. Our finding is in agreement with that of Wang *et al.* [9], who reported the increase in free radical scavenging power of strawberry by elevated CO_2_ concentrations (950 µmol mol^-1^). This study demonstrated that ginger has good free radical scavenging ability and, hence, can be used as a radical inhibitor or scavenger, possibly acting as a primary antioxidant. At the same time, with the anticipated rise in CO_2_ concentrations in the future with the current climate change scenario, it is anticipated that the antioxidant properties of ginger extracts could be enhanced, as the results indicate that an increased atmospheric carbon dioxide concentration could have a major effect on the antioxidant capacities of young ginger varieties. 

## 3. Experimental

### 3.1. Plant Materials

Rhizomes of two varieties of *Zingiber officinale* Roscoe. (Halia Bentong and Halia Bara) were germinated for two weeks in small pots and then transferred to 15 cm by 18 cm polyethylene bags filled with a soiless mixture of burnt rice husk and coco peat in a ratio of 1:1. After 2 weeks, the plants were transferred to a CO_2_ growth chamber (Conviron EF7, Winnipeg, Canada) with two different CO_2_ concentrations. 

### 3.2. Growth Chamber Microclimate

First 400 µmol mol^-1^ was used as ambient CO_2_ condition, and 800 µmol mol^-1^ as the elevated CO_2_ concentration. Pure carbon dioxide (99.8% purity) was supplied from a high concentration carbon dioxide cylinder and injected through a pressure regulator into the closed fumigation chamber. The flow and concentration of carbon dioxide to the chamber was monitored and controlled with a CO_2_ PPM3 Controller. Water being supplied throughout the experiment using a drip system to ensure normal plant growth as water was supplied directly to the root zone of seedlings by pen drippers. Photoperiod, relative humidity, and air temperature of the chamber were controlled using an integrated control, monitoring, and data management system software package (Dynamac Corp., Rockville, MD). Plants were harvested after 16 weeks of CO_2_ exposure, and leaves and rhizomes were separated, freeze dried and finally kept at -80 ºC for future analysis. The experiments were carried out at the Biosystem Laboratory, Engineering Faculty, Universiti Putra Malaysia (UPM). 

### 3.3. High Performance Liquid Chromatography (HPLC)

#### 3.3.1. Flavonoid Extract Preparation

Aliquots of leaves and rhizomes (0.25 g) were extracted with 60% aqueous methanol (20 mL). Six M HC1 (5 mL) was added to each extract to give a 25 mL solution of 1.2 M HC1 in 50% aqueous methanol. Extracts were refluxed at 90 ºC for 2 h. Extract aliquots of 500 μL, taken both before and after hydrolysis, were filtered through a 0.45 μm filter [47].

#### 3.3.2. Analysis of Flavonoids Composition by HPLC

Reversed-phase HPLC was used to assay flavonoid compositions. The Agilent HPLC system (Tokyo, Japan) used consisted of a Model 1100 pump equipped with a multi-solvent delivery system and an L-7400 ultraviolet (UV) detector. The column was an Agilent C18 (5 µm, 4.0 mm internal diameter 250 mm). The mobile phase composed of: (A) 2% acetic acid (CH_3_COOH) and (B) 0.5% acetic acid-acetonitrile (CH_3_CN),(50:50 v/v), and gradient elution was performed as follows: 0 min, 95:5; 10 min, 90:10; 40 min, 60:40, 55 min, 45:55; 60 min, 20:80; and 65 min, 0:100. The mobile phase was filtered under vacuum through a 0.45 um membrane filter before use. The flow rate was 1 mL min^-1^ and UV absorbance was measured at 280-365 nm. The operating temperature was maintained at room temperature [48]. Identification of the flavonoids was achieved by comparison with retention times of standards, UV spectra and calculation of UV absorbance ratios after co-injection of samples and standards. Commercial standards were purchased from Sigma–Aldrich (St Louis, MO, USA).

#### 3.3.3. Preparation of Phenolics Extracts

Phenolics extracts were prepared by first carefully pipetting phosphoric acid (H_3_PO_4_, 1.2 mL) into about 950 mL water in a 1-L volumetric flask, mixing and bringing to volume with water. Then leaves and rhizomes (0.25 g) were extracted with 20 mL, of this phosphoric acid solution. Five mL of 6 M HC1 was added to each extract to give a 25 mL solution of 1.2 M HC1 in 50% MeOH. Extracts were refluxed at 90 ºC for 2 h and solution were filtered through a 0.45 μm filter [49]

#### 3.3.4. Analysis of Phenolics Acids Composition by HPLC

An Agilent HPLC system (Tokyo, Japan) consisting of a Model 1100 pump equipped with a multi-solvent delivery system and a L-7400 ultraviolet (UV) detector was used. The column was an Agilent C18 (5 µm, 4.6 mm internal diameter 250 mm). The mobile phase was composed of phosphoric acid (aqueous) and (B) acetonitrile and gradient elution was performed as follows: 0 min, 85:15; 12 min, 75:25; 20 min, 75:25; 22 min, 85:15 and 30 min, 85:15. The mobile phase was filtered under vacuum through a 0.45 lm membrane filter before use. The flow rate and injection volume were 1 mL min^-1^ and 20 μL. UV absorbance was measured at 220-365 nm. The operating temperature was maintained at room temperature [49]. Identification of the phenolic acids were achieved by comparison with retention times of standards, UV spectra and calculation of UV absorbance ratios after co-injection of samples and standards. Commercial standards were purchased from Sigma–Aldrich.

### 3.4. Determination of Antioxidant Activities

#### Radical Scavenging Assay (DPPH)

1,1-Diphenyl-2-picrylhydrazyl (DPPH) was purchased from Sigma–Aldrich. Butylated hydroxytoluene (BHT) and α-tocopherol were purchased from Merck (India). In order to determine the radical scavenging ability, the method reported by Mensor *et al.* [50], was used. Briefly, an alcohol solution of DPPH (1 mL, 3mg/mL) was added to 2.5 mL samples containing different concentrations of extracts originating from different parts of the ginger varieties. The samples were first kept in a dark place at room temperature and their absorbance was read at 518 nm after 30 min. The antiradical activity (AA) was determined using the following formula: 


AA% = 100- ((Abs: sample - Abs: empty sample) × 100)/ Abs: control

Blank samples contained 1 mL ethanol + 2.5 mL of various concentrations of ginger extract; control sample contained 1 mL of 0.3 mM DPPH + 2.5 mL ethanol. The concentration of the samples, the control, and the empty samples were measured in comparison with ethanol. The synthetic antioxidants BHT (butylated hydroxytoluene) and α-tocopherol were used as positive controls.

### 3.5. Statistical Analysis

The experiment was split-split plot and results were expressed as mean ± standard deviation (SD). Where applicable, the data were subjected to one way analysis of variance (ANOVA) and the differences between samples were determined by Duncan’s Multiple Range test using the Statistical Analysis System (SAS, 1999) and MSTATC program. P values ≤ 0.05 were regarded as significant.

## 4. Conclusions

The results of current study indicate that the synthesis of phenolics and flavonoids in ginger can be increased and affected by using CO_2_ enrichment in a controlled environment (CE). Following that, the antioxidant activity in young ginger extracts could also be improved. The HPLC analyses of flavonoids and phenolic compounds of CO_2_-enriched ginger plants also exhibited the ability of such treated plants to synthesize new compounds such as vanillic acid, cinnamic acid and salicylic acid. According to previous studies salicylic acid can enhance plant growth. Among the flavonoid compounds synthesized, fisetin and kaempferol, together with the phenolic compounds gallic acid and vanillic acid were dramatically enhanced in ginger parts by the elevated CO_2_ enrichment. This is a first report of fisetin, morin and naringenin in young ginger. Increases of these components in the rhizomes were more than in the leaves, and this was attributed to simultaneous improvement of antioxidant activities in rhizomes of those varieties when grown under elevated carbon dioxide conditions. This is a significant finding because the practice of CO_2_ enrichment has the potential to increase the value of the chemical product per unit area, and increase bio-activity per gram dry matter, emphasizing the implication on the phytochemistry of valuable medicinal and food plants such as in *Zingiber officinale.*

Natural antioxidants, especially phenolics and flavonoids from tea, wine, fruits, vegetables, and spices are already exploited commercially, either as antioxidant additives or as nutritional supplements. In recent years many plant species have been investigated in the search for novel antioxidants, but generally there is still a demand to find more information concerning the antioxidant potential of plant species as they are safe and also bioactive. The results on antioxidant activity of ginger indicate a good potential in this area for both the rhizomes as well as the leaves, especially when grown under elevated carbon dioxide. On the other hand, the impacts of cultural conditions and CO_2_ concentration on biopharmaceutical production in herbs have not been widely investigated and it needs to be understood, especially when the objective is the optimization of the herb chemistry. From this work, it can also be suggested that the composition of the phenolics and flavonoids will have to be considered in the development of any CE production system for medicinal plants. Further work is required to confirm this suggestion. 
